# Colorimetric Sensing of Amoxicillin Facilitated by Molecularly Imprinted Polymers

**DOI:** 10.3390/polym13132221

**Published:** 2021-07-05

**Authors:** Joseph W Lowdon, Hanne Diliën, Bart van Grinsven, Kasper Eersels, Thomas J. Cleij

**Affiliations:** Sensor Engineering Department, Faculty of Science and Engineering, Maastricht University, P.O. Box 616, 6200 MD Maastricht, The Netherlands; hanne.dilien@maastrichtuniversity.nl (H.D.); bart.vangrinsven@maastrichtuniversity.nl (B.v.G.); kasper.eersels@maastrichtuniversity.nl (K.E.); thomas.cleij@maastrichtuniversity.nl (T.J.C.)

**Keywords:** amoxicillin, emulsion polymerization, molecularly imprinted polymers, colorimetric sensor, displacement assay

## Abstract

The scope of the presented research orientates itself towards the development of a Molecularly Imprinted Polymer (MIP)-based dye displacement assay for the colorimetric detection of the antibiotic amoxicillin in aqueous medium. With this in mind, the initial development of an MIP capable of such a task sets focus on monolithic bulk polymerization to assess monomer/crosslinker combinations that have potential towards the binding of amoxicillin. The best performing composition (based on specificity and binding capacity) is utilized in the synthesis of MIP particles by emulsion polymerization, yielding particles that prove to be more homogenous in size and morphology compared to that of the crushed monolithic MIP, which is an essential trait when it comes to the accuracy of the resulting assay. The specificity and selectivity of the emulsion MIP proceeds to be highlighted, demonstrating a higher affinity towards amoxicillin compared to other compounds of the aminopenicillin class (ampicillin and cloxacillin). Conversion of the polymeric receptor is then undertaken, identifying a suitable dye for the displacement assay by means of binding experiments with malachite green, crystal violet, and mordant orange. Once identified, the optimal dye is then loaded onto the synthetic receptor, and the displaceability of the dye deduced by means of a dose response experiment. Alongside the sensitivity, the selectivity of the assay is scrutinized against cloxacillin and ampicillin. Yielding a dye displacement assay that can be used (semi-)quantitatively in a rapid manner.

## 1. Introduction

Since the discovery of modern-day antibiotics in the early 20th century, these miracle molecules have gone from a wonder drug to one of the world’s largest environmental contaminants [1]. Over the last couple of decades, the consumption and use of antibiotics has increased, not just by humans but also by cattle [2]. One of the biggest consumers of antibiotics is China, with a reported use of 162,000 tons of antibiotics in 2013, with more than half of which administered to animals [3]. It is common practice to supplement animal feed with antibiotics, ensuring the health and welfare of the creature by reducing the risk of illness or facilitating the rapid recovery from bacterial ailments [4]. Though this is beneficial for the animal in question, it generates an environmental concern. Incomplete metabolism of the antibiotics leads to 30–90% of the active compounds being excreted by the animal, and in the process introducing the antibacterial agents to their surrounding environment [5,6]. Thus, the concentrations of antibiotics in the environment is increasing, and the bacteria that are exposed consistently to these compounds become resistant to their destructive effects. This is just one concern related to the over-use of antibiotics in animals, another is the transfer of antibiotics in animal-related byproducts, e.g., milk [7]. This makes the detection of antibiotics in both animal waste and in by-products essential, as both the environment and animals (humans) are at risk due to the potential of stimulating and further progressing antibiotic-resistance.

Molecularly imprinted polymers (MIPs) are a developing technology that has shown promise in the field of sensing of low molecular weight compounds, and have demonstrated great potential in the analysis of samples contaminated with antibiotics [8,9]. MIPs are synthetic polymeric receptors containing nanocavities that have been specifically tailored towards the binding of a chosen molecule, making them the artificial equivalent to antibodies [10,11,12]. The benefit over their biological counterparts lies in the stability of the receptors, demonstrating high resistance to harsh physical conditions such as pH and temperature while retaining sensitivity [13]. This makes them ideal for the analysis of matrices that are outside the normal physiological conditions (wastewater, soil samples) that would normally render the biological receptors useless. To this end, MIPs have been developed in the analysis of antibiotics in the environment, coupling the synthetic receptors to various transducer (e.g., Quartz Crystal Microbalance (QCM), thermal methods, and fluorescent probes) translating binding events at the surface of the MIP into a tangible quantifiable signal [14,15,16,17,18,19]. Though these technologies for the analysis and sensing of antibiotics in complex samples are rapidly progressing, these readout techniques still require a level of expertise to conduct (though great efforts have been made to increase the portability of devices and to make them as user-friendly as possible). A more simplistic MIP-based variation is possible, developing the MIP into a dye displacement assay for the rapid visual verification of a target’s presence [20].

The use of MIPs in conjunction with dyes was first conceived in 1998 by McNiven et al. They used a dye-conjugated analogue of chloramphenicol in a competitive capacity to detect the presence of the parent compound [21]. Introduction of the parent compound to the system would displace the dye-conjugate in a quantitative manner, yielding higher displacement of the conjugate in direct correlation to the amount of chloramphenicol present. This concept would take many other forms over the next two decades, modifying template molecules to include chromophores and fluorophores, introducing optical properties into the structures of the MIPs, and demonstrating that the structure of a pre-bound signaling molecule could share no relation to the desired target analyte to be displaced [22,23,24,25]. The premise was recently extended further by using commercially available dyes such as malachite green and crystal violet, developing MIP-based dye displacement assay for the detection of diarylethylamines and amphetamine [26,27]. The research highlighted how low-cost visual sensors can be engineered from MIP technology, though for relatively simple molecules with few competing functionalities. Aminopenicillins, however, are not simple molecules.

**Figure 1:**
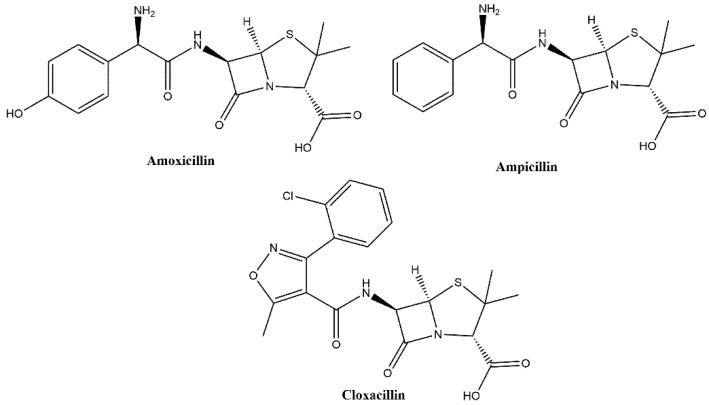
The chemical structures of amoxicillin, ampicillin, and cloxacillin.

Showing a diverse molecular structure, aminopenicillins encompass a broad range of structural derivatives of ampicillin (which itself is an amino benzylpenicillin) (Figure 1) [28,29]. The multiple functionalities within the molecules gives rise to the possibility of different binding opportunities, meaning the selection of functional monomers/crosslinkers is not as definitive as for simpler mono-functional molecules [30]. Therefore, a degree of rational design is required, analyzing the potential binding groups and selecting compositions that not only interact with one functional, but with multiple [31]. The same principles can be applied to the selection of a dye for conversion of a MIP into a dye displacement assay. Enabling a comparison of the key functionalities between dye molecule and template molecule, thus optimizing the binding and displacement of the dye. However, optimized ratios and chemical interactions are only one part of the story, as further optimization is possible by improving the polymerization method employed to synthesize said MIPs.

The most common method of producing MIPs is monolithic bulk imprinting, where all components of a MIP are placed into a single container and polymerized [32]. The resulting monolith can be crushed and the template extracted, yielding a powder with a crude morphology [33]. This protocol is robust and can enable the rapid testing of polymer formulations when attempting to design an MIP [34]. Although the bulk imprinting approach is a tried-and-tested approach of successfully producing MIPs, the methodology has been surpassed by more elegant approaches that enable more homogenous morphologies and particles sizes [35,36,37,38,39,40]. One such methodology is emulsion polymerization, where two phases (aqueous and organic) are introduced in the presence of a surfactant that enables the formation of uniformly sized micelles [41]. These act as micro-reactors containing the polymerization mixture, which in turn enables the formation of more homogenous morphologies. Though more elegant, this kind of methodology lacks in simplicity when compared to its more crude predecessor.

With all this in mind, this research builds upon prior literature, taking optimized monomer/crosslinker ratios and expanding these ratios towards novel compositions for the binding of amoxicillin. Monolithic bulk imprinting is used to rapidly test these compositions, before synthesizing MIPs with higher homogeneity by an emulsion-based approach. The resulting MIP is compared to its monolithic bulk imprinted counterpart, comparing imprint factors at different free concentrations of target analyte. Scrutiny is drawn towards the selectivity of the emulsion-made MIP, before determining the binding of a variety of dyes (malachite green, crystal violet, and mordant orange 1) that could potentially be used in the dye displacement assay format. The optimum dye was consequently selected by comparing binding affinities and capacities with that of amoxicillin, ensuring there was a great enough difference in these values to facilitate displacement. Finally, the best performing dye is loaded onto the emulsion MIP by means of incubation and the assay format analyzed. To this end, dose response and selectivity tests are conducted, determining the sensitivity of the assay towards amoxicillin and seeing how the assay fairs in the presence of other antibiotics of the same class (cloxacillin and ampicillin). Overall, the research highlights how pragmatic compositional choices, alongside intelligent dye selection can be used to produce an MIP that can be easily developed into a semi-quantitative rapid colorimetric assay format.

## 2. Materials and Methods

### 2.1. Chemicals and Equipment

Acrylamide (AA, 99.5%, CAS: 79-06-1), Methacrylic acid (MAA, 99.5%, CAS: 79-41-4), ethylene glycol dimethacrylate (EGDMA, 99.5%, CAS: 97-90-5), trimethylolpropane trimethacrylate (TRIM, CAS: 3290-92-4), dimethyl sulfoxide (DMSO, reagent grade, CAS: 67-68-5), Malachite green oxylate salt (99.8%, CAS: 2437-29-8), crystal violet (99.8%, CAS: 548-62-9), mordant orange 1 (70%, CAS: 2243-76-7), aluminum oxide (basic, CAS: 1344-28-1), amoxicillin (99.8%, CAS: 26787-78-0), cloxacillin (99.8%, CAS: 61-72-3), and ampicillin sodium salt (99.5%, CAS: 69-52-3) were purchased from Sigma-Merck. Prior to polymerization, all stabilizers were removed from monomers and crosslinkers by passing the compounds over aluminum oxide (basic), thus removing any chemical source that could inhibit the polymerization process. Equipment utilized in the experimentation include a Shimadzu-3600 UV-Spectrophotometer (3.5 mL quartz cuvette, path length of 1 cm), BlueWave 200 UV light source, Leica Microsystem DM300, and a Shimadzu MIRacle10 FT-IR spectrometer equipped with a Zn/Se ATR crystal over an optical range of 4000 to 450 cm^−1^.

### 2.2. Generalised Monolithic Bulk Imprinting Method

The component ratios used for the MIPs synthesized were based on previous literature regarding the monolithic polymerization of molecularly imprinted polymers [42]. In general, functional monomer (1.4 mmol), template (0.35 mmol), crosslinker (2.8 mmol), and DMSO (3.3 mL) were placed in a glass vial and dissolved O_2_ purged from the mixture by bubbling N_2_ through the solution for 30 s. AIBN (36.1 mg, 0.22 mmol) was then added to the resulting mixture, ensuring that the solution was thoroughly mixed before subjecting the solution to a UV lamp (BlueWave 200, wavelength: 300–450 nm), thus initiating polymerization under photochemical conditions. The polymeric mixture was exposed to the UV lamp for 30 min, before yielding a solid polymer block. The resulting monolith proceeded to be mechanically ground with a ball mill (3 cycles, 300 rpm for 180 s, 60 s pause between each cycle), producing a fine powder with an average particle size less than 150 μm (confirmed by microscopy). After mechanical grinding, the powder was subject to continuous soxhlet extraction with ethanol/acetic acid (100:1) for 24 h, before further extraction with ethanol for 12 h and drying the powder. The same process was completed excluding the template molecule generating a non-imprinted polymer (NIP) that would act as a reference material. The composition of the MIPs/NIPs synthesized can be found in Table 1.

### 2.3. Generalized MIP Synthesis by Emulsion Polymerization

**Figure 2:**
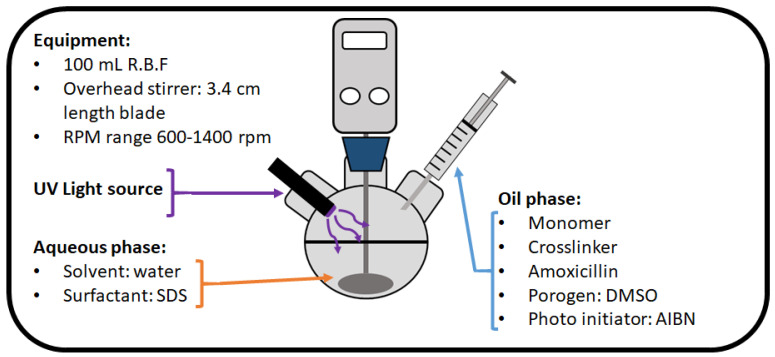
Apparatus schematic for the emulsion polymerization approach of synthesizing MIPs/NIPs with more uniform particle sizes.

An aqueous phase was prepared by mixing 300 mg of sodium dodecyl sulphate (SDS) in 60 mL of DI (deionized) water and placing it into a 100 mL round bottom flask. An overhead stirrer was then placed into the flask, with the stirrer blade positioned so that it was below the surface of the solution. Proceeding this, the organic phase of the reaction was prepared by mixing monomer (MAA/AA), EGDMA, AIBN, amoxicillin, and DMSO (compositions in Table 1) in a vial before purging both solutions with N_2_ for 30 s. Once purged, the organic phase was introduced dropwise to the aqueous phase while being vigorously stirred between 600–1400 rpm for 1 min and enabling an emulsion to form (see Figure 2). Stirring was ceased, and the emulsion exposed to UV light (BlueWave 200) for 1 h. After the exposure, the solution was transferred to centrifuge tubes and spun at 4500 rpm for 5 min. The resulting polymeric disk was left in the centrifuge tube while carefully removing the supernatant and introducing ethanol to the tube. The tube was shaken well suspending the polymeric powder and the centrifugation process repeated for five washes with ethanol. Once fully washed the polymeric powder was transferred into a glass vial and oven-dried at 65 °C for 12 h. Thus yielding a fine polymeric powder with a defined particle size based on the rpm initially used to mix the reaction. A non-imprinted reference version of the polymeric composition was synthesized with the exact same methodology described, but without the presence of the template molecule. Template extraction was monitored with FTIR comparing the non-extracted MIP, the extracted MIP, and the amoxicillin spectra. Particle sizes were analyzed by optical microscopy (Leica Microsystem DM300), while the 1 mg mL^−1^ of polymer particles were suspended in water.

### 2.4. Generalised Batch Rebinding Experiment

To determine the binding affinities of the MIP/NIP, rebinding experiments were conducted as follows: to 20 mg of MIP/NIP powder was added 5 mL aliquots of aqueous target/analogue ranging in concentration (0.1–0.7 mM) and the resulting suspension agitated on a rocking table (125 rpm) for 90 min. After agitation, the filtrate (0.45 µm pour size, PTFE filters, VWR, cat number: 514-0065) of each sample was collected and analyzed using a UV-spectrophotometer, analyzing the λ_max_ of the remaining molecular species in solution (C_f_). The amount of molecular species bound to the MIP/NIP (S_b_) proceeded to be calculated from these observed values and the corresponding binding isotherm plotted. This process was repeated for each of the following species: Amoxicillin, ampicillin, cloxacillin, malachite green, crystal violet, and mordant orange 1.

**Note:** During the experimentation with malachite green, all samples of crystal violet and mordant orange were diluted (dilution factor 100), ensuring absorbance values within the reliable range on the UV-spectrophotometer.

### 2.5. Preloading of Dye to MIPs

A total of 1 g of MIP 205 powder was added to 50 mL of aqueous mordant orange (1 mM). The resulting suspension was well stirred for half an hour before transferring the suspension into a centrifuge tube. The solution was then centrifuged at 4500 rpm for 5 min, enabling the safe removal of the supernatant, while allowing the MIP pellet to remain at the bottom of the tube. Proceeding this, 45 mL of DI water was added to the tube, the vessel sealed, and the tube shaken thoroughly, facilitating the dispersal of the MIP powder once more. The centrifugation process was then repeated and the water changed until the resulting supernatant appeared colorless and showed no signs of a residual dye absorbance band by means of UV-spectrophotometry.

### 2.6. Dose Response of Dye Displacement

A dose-response study was constructed by incubating the synthesized dye loaded MIP 205 (40 mg) with 5 mL of aqueous amoxicillin at varying concentrations (0.000–365.0 mg L^−1^) for 1 min before the filtration (0.45 µm pour size, PTFE filters, VWR, cat number: 514-0065) of the sample. The filtrate proceeded to be analyzed by a UV-spectrophotometer collecting the spectrum for each sample (200–500 nm). The maximum absorbance of each concentration (λ_max_ = 385 nm) was then plotted against the concentration of amoxicillin added, allowing the dose response to graphed. The same process was repeated for the assay in the presence of cloxacillin and ampicillin, testing the selectivity of the assay.

## 3. Results

### 3.1. Analysis of Bulk MIPs

**Figure 3:**
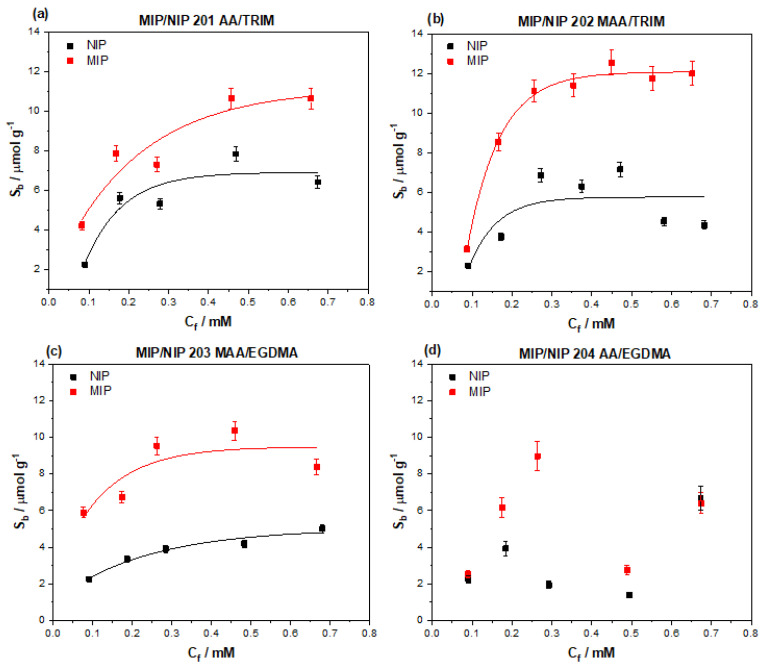
Binding isotherms for the varying MIP compositions (**a**) AA/TRIM, (**b**) MAA/TRIM, (**c**) MAA/EGDMA, and (**d**) AA/EGDMA synthesized by monolithic free radical polymerization.

Four initial MIP compositions were selected for the binding of amoxicillin after identifying the amine and carboxylic acid functionalities as ideal groups for possible ionic and hydrogen bonding interactions. The four compositions consisted of either methacrylic acid, acrylamide (targeting different chemical functionalities within the amoxicillin), and either EGDMA or TRIM as crosslinker (EGDMA producing a more flexible structure, and TRIM a more rigid network). The binding properties of the MIPs were tested by means of batch rebinding experimentation, where the MIPs were exposed to increasing concentrations (0.1–0.7 mM) of amoxicillin for a defined time (90 min). After the incubation period the MIPs were removed from the solutions and the remaining concentration of amoxicillin in solution determined (C_f_), thus the amount of bound amoxicillin to the MIP could be realized (S_b_). These values were then plotted against each other, yielding the binding isotherm for each MIP composition (Figure 3).

The plotted data points were then fit using an allometric function (y = ax^b^) factoring in the morphology of the polymeric structure and therefore surface area, simulating a saturation effect as higher concentrations of molecular species are introduced to the MIP/NIP. MIP 201–203 clearly demonstrate this saturation effect towards the higher concentrations, whereas MIP 204 shows no correlation. Of the MIPs tested, MIP 202 has the highest perceived binding capacity (S_b_) of 12.10 μmol g^−1^, indicating it has the highest affinity of all the MIPs tested towards amoxicillin. MIP 201 and MIP 203 have slightly lower binding capacities of 10.72 and 9.47 μmol g^−1^, respectively, whereas MIP 04 has an incalculable binding capacity and no feasible trend exists within the data points collected.

A metric was placed on how specific the binding interactions are between the MIP and target molecule by comparing the S_b_ of the MIP and NIP at a defined free concentration (in this case C_f_ = 0.1, 0.2, and 0.3 mM), yielding a value known as the imprint factor (IF) describing the specific interactions of the MIP with the target (Table 2). The MIP with the highest amount of specific interaction was MIP 203, demonstrating IF values of 2.55 (C_f_ = 0.1 mM), 2.48 (C_f_ = 0.2 mM), and 2.33 (C_f_ = 0.3 mM). The IF values calculated for MIP 201 and 202 across this range are diminished in comparison, with MIP 201 having the lowest specific interaction out of the two. The performance of MIP 202 is observed to increase over the calculated concentration range, though the generated IF values are still lower than those calculated for MIP 203.

**Figure 4:**
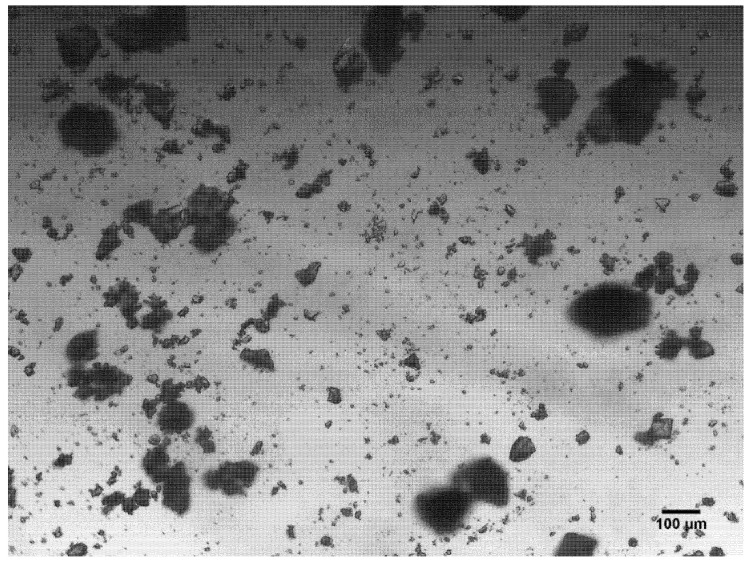
Optical microscope images of finely ground bulk monolith particles below 150 μm in size.

Following the binding analysis of the MIPs, the morphology of the particles was brought into question. Optical microscope images of the particles were taken allowing an assessment of the homogeneity of particle sizes and shapes to be conducted (Figure 4). The images reveal the morphology of the particles is inconsistent and shows a large degree of heterogeneity; therefore, each particle has differing surface area and different potential to interact with molecular species.

### 3.2. Emulsion Particle Size Analysis

**Figure 5:**
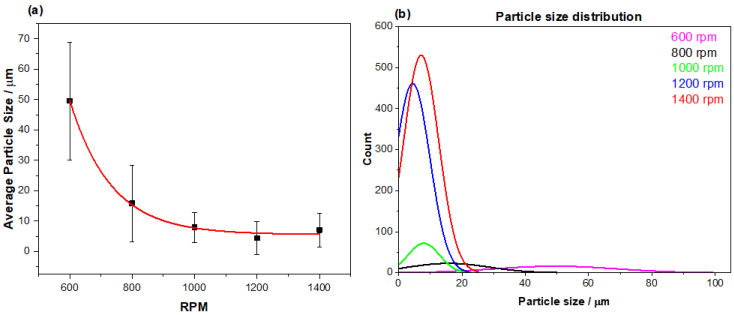
How the stirring speed effects the particle size generated during the emulsion polymerization process with (**a**) relating the average achieved particle sizes with the rpm used (error bars being the ±1 standard deviation in the size measurements), and (**b**) the distribution of the particle sizes at each speed.

In an attempt to generate more homogenous MIP particle sizes, an emulsion polymerization approach was adopted. To this end, the most promising monomer and crosslinker combination from the bulk experiments was brought forward (MIP 203), incorporating the same stoichiometric ratios in the emulsion polymerization process. The methodology entailed the addition of an organic phase (monomer, template, crosslinker, porogen, and initiator) to an aqueous phase (water and surfactant), thoroughly mixing the two phases to produce an emulsion that could yield finer more homogenous particle sizes (further details in discussion). The degree to which the two phases were mixed was investigated, relating particle size to the speed of the stirring invoked (Figure 5a). Plotting the average particle size produced against the speed (rpm) at which the overhead stirrer was set revealed that the slower (600 rpm) produced larger particles with a higher standard deviation in size (50 ± 19 μm) than at the higher speeds. As the stirring surpassed 1000 rpm, the particle sizes achieved began to plateau, reaching an average particle size of 8–10 μm with a standard deviation range of ± 3–4 μm. The relationship between the lower standard deviation in relation to the speed of stirring is strengthened by the analysis of the particle size distribution (Figure 5b), which indicates the lower speeds produce a wider spread of particle sizes compared to higher speeds producing a narrower band of particle sizes.

**Figure 6:**
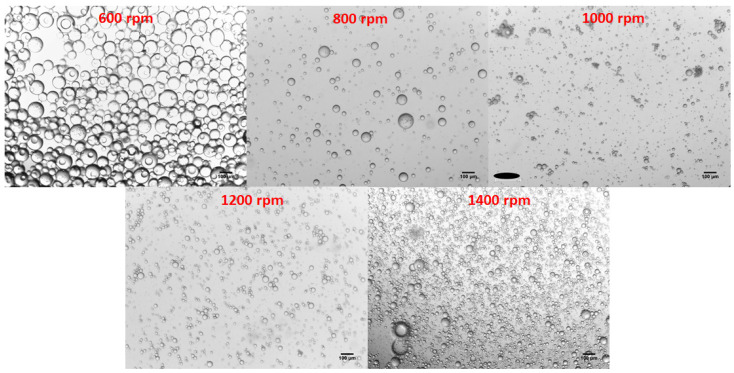
Optical microscope (Leica Microsystem DM300) images of particles sizes produced by varying the stirring speed during the emulsion polymerization process.

Alongside the particle size distributions, the morphology of the particles at each stirring speed was also assessed (Figure 6). Though the size of the particles may differ with the varying speeds, the overall morphology of the particles remained consistent throughout. Each emulsion produced spherical morphologies with very little deviation in structure apart from the size differences observed.

### 3.3. Emulsion MIP Binding Analysis

**Figure 7:**
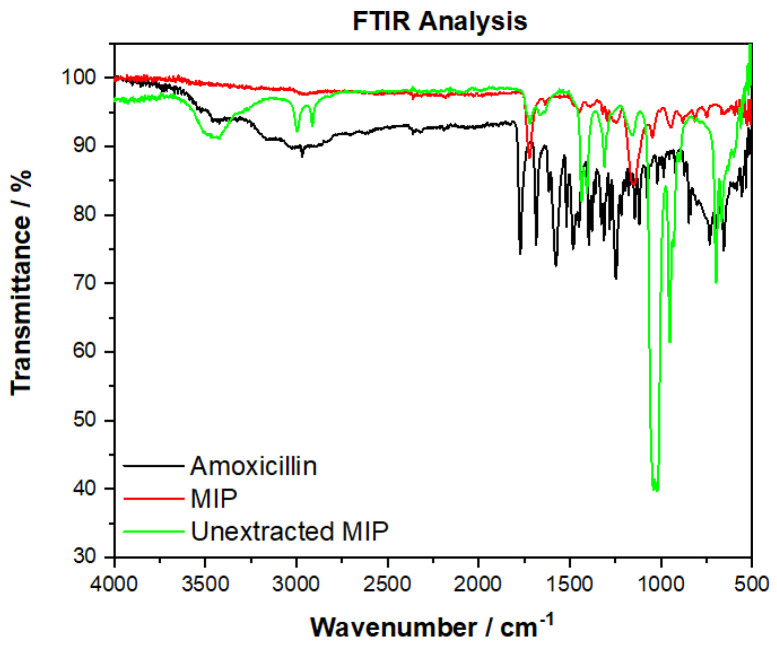
FTIR analysis of amoxicillin (black line), unextracted MIP (green line), and the extracted emulsion MIP (red line), demonstrating that amoxicillin was successfully removed from the imprinted material.

To ensure the washing procedure successfully removed the template amoxicillin, an FTIR comparison of the extracted MIP was compared to that of the non-extracted MIP and amoxicillin (Figure 7). The amoxicillin has very distinctive peaks between 2500–3500 cm^−1^ (OH stretch) that are not present in the extracted MIP, but are present in the non-extracted MIP. Other key regions that relate to the structure of amoxicillin (e.g., sharp peaks between 1680 cm^−1^ (C=O, amide), 1390–1310 cm^−1^ (O-H bending, phenol), and below 900 cm^−1^) are not present in the analysis of the MIP, therefore it can be said with confidence that amoxicillin is no longer present in the extracted imprinted polymer. Comparing non-extracted with extracted, the sulfoxide peak (S=O, 1070–1030 cm^−1^) is seen to disappear, suggesting the washing step is also successful in removing unwanted porogen from the polymer.

**Figure 8:**
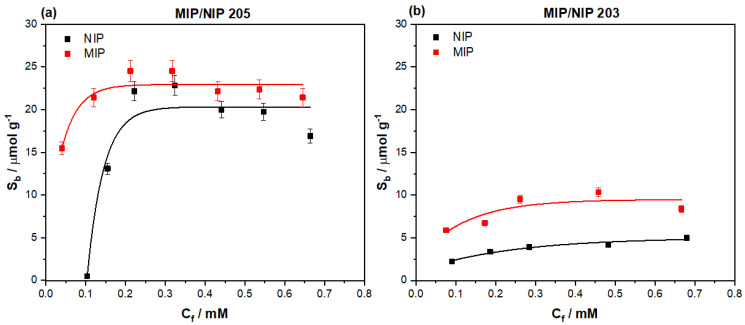
Binding isotherms for (**a**) MIP 205 prepared by emulsion polymerization and (**b**) a rescaled MIP 203 prepared by bulk polymerization strategies, where both MIP (red squares) and NIP (black squares) were plotted and fit.

After ensuring the template molecule was fully extracted from the MIP, the same rebinding procedure was followed as with the bulk generated MIP particles. The MIP 205 particles were incubated with increasing concentrations of amoxicillin, analyzing the remaining amount of amoxicillin left in solution after the binding process. Thus, the amount of template bound to the MIP (S_b_) was plotted against the remaining free concentration (C_f_) of amoxicillin in solution (Figure 8a). The data was fit using OriginPro8 (OriginLabs Corporation, Northampton, MA, United States) using an allometric (y = ax^b^) fit, modelling the binding isotherm of both the MIP (red line, R^2^ = 0.84058) and NIP (black line, R^2^ = 0.9173).

This data was directly compared to the binding isotherm of the monolithic bulk polymerized MIP 203, rescaling the binding isotherm’s *y*-axis (S_b_) to enable the direct comparison of the features of the isotherms (Figure 8b). The emulsion MIP demonstrated a higher overall binding capacity of 22.53 μmol g^−1^ once saturation (plateauing) is observed, comparatively, the monolithic MIP only demonstrated a binding capacity of 9.89 μmol g^−1^. At lower C_f_ values (0.05–0.1 mM) the emulsion prepared MIP 205 particles exhibit a high affinity to the amoxicillin with little to no interaction with the corresponding NIP, unlike the monolithic bulk NIP, where binding at the lower concentrations is apparent. MIP 203 consequently performed consistently throughout the concentration range, showing a steady trend of binding the amoxicillin in a similar manner at both high and low concentrations in comparison with the NIP.

For a more direct comparison of the specific nature of the MIPs, the imprint factor (IF) was calculated for MIP 205 and compared to that of MIP 203 (Table 3). The comparison of this metric shows that MIP 205 interacts vastly more specifically at this low concentration compared to MIP 203. MIP 205 has a calculated IF (C_f_ = 0.1 mM) of 45.46 compared to that of MIP 203 that has an IF = 2.55 (C_f_ = 0.1 mM). Considering these findings, the research will focus on the use of the MIP at low concentrations analyte when incorporating the synthetic receptor in the developed dye displacement assay.

**Figure 9:**
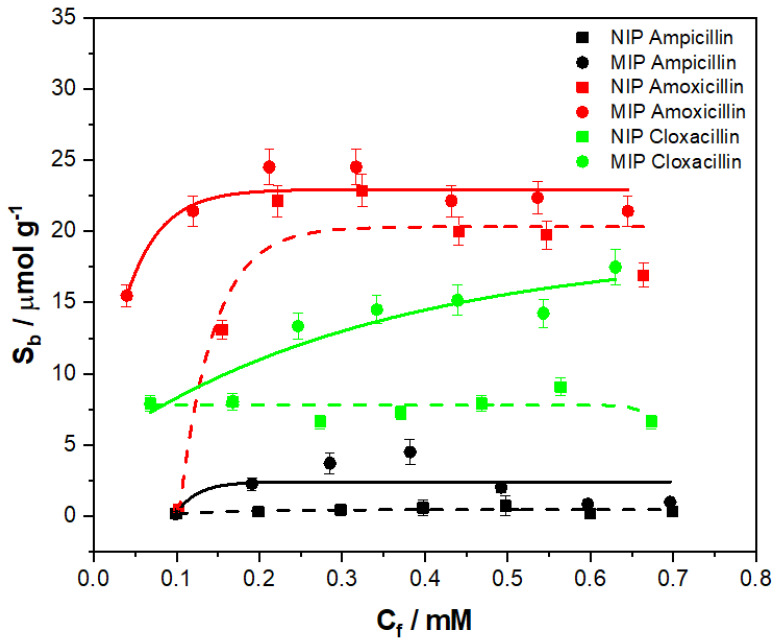
Binding isotherm for the interaction of ampicillin (black squares/circles), cloxacillin (green squares/circles), and amoxicillin (red squares/circles) with MIP 205, where the interactions of both MIP (circles) and NIP (squares) are displayed.

The specificity of a MIP is not the only facet of value when determining an MIPs performance, the selectivity also plays a major role in determining an MIP’s potential. Therefore, the selectivity of the MIP 205 was scrutinized by performing binding experiments with analogues of amoxicillin. For this, the aminopenicillins ampicillin and cloxacillin were selected, being similar in chemical structure and in the same class of compounds to that of amoxicillin. The binding experiment was conducted in the same manner as previously outlined, ensuring the concentrations of the aminopenicillin analogues was in the same range (0.1–0.7 mM), the mass of MIP/NIP utilized (20 mg) was constant and the incubation period remained the same. The corresponding binding isotherms were then plotted, and allometrically fitted (y = ax^b^) for comparison (Figure 9).

Of the analogues tested, cloxacillin proved to have the higher level of interaction in terms of binding capacity with the MIP/NIP 205 out of the two derivative compounds. The interaction with the MIP (green circles) gradually increased with the C_f_, with the allometric fit (green line, R^2^ = 0.81599) describing the data relatively well and yielding a maximum binding capacity (S_b_) of 16.43 μmol g^−1^. The interaction of cloxacillin with the NIP (green squares) was also consistent at purveying a binding average S_b_ of 7.61 μmol g^−1^. In comparison, the binding for the ampicillin (Figure 9 was much lower with the MIP (black line, R^2^ = 0.28487) yielding a maximum S_b_ of 4.71 μmol g^−1^ and the NIP (black dashed line, R^2^ = 0.29573) a maximum of 0.53 μmol g^−1^. When comparing the maximum binding capacities of the compounds against that of amoxicillin, it is clear that they are lower. However, further analysis of the selectivity of the MIP can be conducted by calculating IF values for each of the derivatives for a direct comparison of the specific interactions.

As with the monolithic bulk polymerization MIPs, the IF was compared at the same C_f_ values (C_f_ = 0.1), enabling a comparison of the specific binding interactions (Table 3). The amoxicillin demonstrates the highest specific interaction with the MIP, with ampicillin having the higher level of specific interaction out of the two analogues.

### 3.4. Selection of Dye

**Figure 10:**
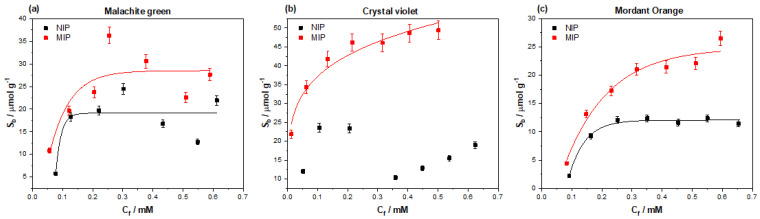
The binding isotherms outlining the interactions of (**a**) malachite green, (**b**) crystal violet, and (**c**) mordant orange with MIP/NIP 205. The plotted values for each of the binding isotherms were extrapolated from the absorbance peaks for each compound at their λ_max_ values (616, 590, and 385 nm respectively), enabling C_f_ values to be calculated and subsequently S_b_ values.

**Figure A1:**
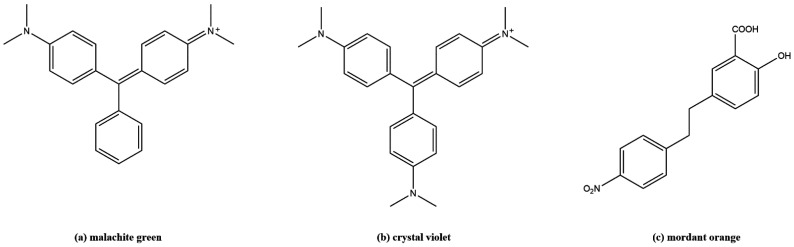
The chemical structure of (**a**) Malachite green, (**b**) Crystal violet, and (**c**) Mordant orange.

To develop the MIP into a displacement assay capable of producing a visual confirmation for the presence of amoxicillin in solution, different dye molecules must be considered for the application in the assay. Considering the chemical composition of the MIP being used to detect the amoxicillin (methacrylic acid/ethylene glycol dimethacrylate co-polymer) dye molecules that can form ionic and hydrogen bonding interactions with the acidic functionalities present were selected. Malachite green, crystal violet, and mordant orange have either amine functionalities (malachite green/crystal violet) or acidic functionalities (mordant orange) capable of forming these interactions (see Appendix A, Figure A1, for chemical structures). The binding interactions of these species with MIP 205 was therefore investigated by means of binding experiments identical to ones discussed prior (Figure 10). The dyes performance was assessed on the maximum binding capacity of the dye compound towards the MIP and the specific interaction (difference between MIP/NIP) exhibited.

Of the dyes tested against the MIP, crystal violet was found to hold the highest affinity towards the polymer. The MIP demonstrated a maximum S_b_ of 49.73 μmol g^−1^ towards crystal violet, being higher than that of affinity towards the amoxicillin template. The same was true for malachite green, with the MIP showing a maximum binding capacity of 28.33 μmol g^−1^, which is once again higher than the observed values for amoxicillin. Mordant orange, however, was found to have a maximum binding capacity of 24.18 μmol g^−1^, which is closer to that of the template molecule. The specific nature of the interactions to the MIPs was once again calculated by dividing the S_b_ of the MIP by that of the NIP at a defined C_f_ (in this case C_f_ = 0.1 for direct comparison with other values calculated), and the values can be found in Table 4.

Due to the nature of the plotted data for crystal violet, there was no reasonable observed line of best fit for the NIP data. The lack of line of best fit therefore made any possible IF calculation for crystal violet invalid and non-comparable to the other dye molecules. Malachite green and mordant orange could, however, have their IF calculated, with mordant orange out performing malachite green. Mordant orange proved to have the higher specific interaction with the MIP with an IF value of 1.94, compared to that of malachite green, which was 1.25.

When considering the overall binding capacity of each dye and the associated IF values, it made most logical sense to select mordant orange to carry forward to the dye displacement assay. The binding capacity of mordant orange reflects a more similar value to that of amoxicillin, whereas the overall binding capacity of malachite green is marginally higher, so may perform less well in the developed assay (further explanation in discussion section).

### 3.5. Dye Displacement

The essence of the dye displacement assay relies on the loading of the reporting dye onto the MIP. This was conducted according to the experimental section in accordance to prior literature [26,27], where a concentrated solution of dye (1 mM) is incubated with the MIP powder (1 g) for a defined period (30 min). After the incubation period is over, the now colored MIP powder is filtered and washed with water until the filtrate has no observable color and can be confirmed by UV-spectrophotometry. Based on the previous experimentation, mordant orange was selected to be preloaded onto the MIP to generate the dye displacement assay.

**Figure 11:**
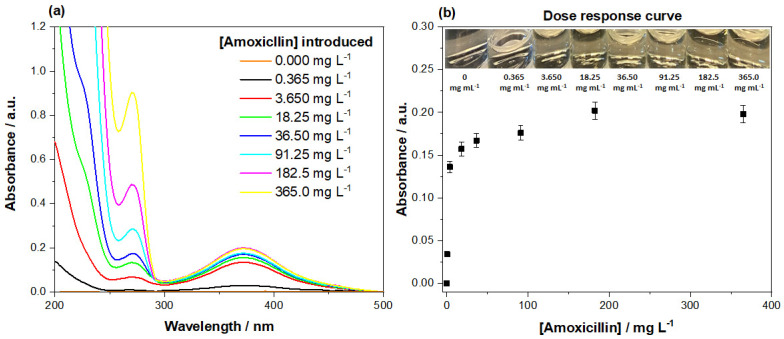
(**a**) The UV absorbance spectra of the filtrate collected from the incubation of differing concentrations of aqueous amoxicillin with the developed dye-loaded MIP, accompanied by (**b**) the plotted dose response correlating to the absorbance (λ = 385 nm) of dye released and the concentration of amoxicillin present.

Once the mordant orange dye was loaded onto the MIPs, a dose response was conducted, correlating displaced dye with the concentration of amoxicillin present (Figure 11a). This was achieved by incubating mordant orange-loaded MIP 205 with varying concentrations of amoxicillin (0.000–365.0 mg L^−1^), ensuring that the volumes added (5 mL), masses (40 mg) used, and incubation time (1 min) remained constant throughout the experiment. The analysis was conducted inside glass vials for ease of use, with disposable syringes and filters (pore size 0.45 µm) used to limit potential contamination from external sources. The dose response was conducted between 0.000–365.0 mg L^−1^ of amoxicillin, in an attempt to determine the minimum concentration required to stimulate the displacement of dye that could be quantified. The spectra clearly shows as the amount of amoxicillin increases in the solution (λ = 270 nm), so does the amount of dye displaced into the medium (λ = 385 nm), and the lowest value plotted (0.365 mg L^−1^) still stimulates a quantifiable response.

The absorbance of the mordant orange (λ = 385 nm) proceeded to be plotted against the concentration of amoxicillin present in the samples (Figure 11b), generating the final dose response graph. The data shows a clear trend, gradually plateauing as the concentration of amoxicillin increases. The initial response is quite sensitive, showing a sharp increase in dye displaced between 0.365 and 3.650 mg L^−1^. After this, the response rapidly diminished, with little to no difference in response to concentrations greater than 91.25 mg L^−1^. Alongside the collect spectra, photos were taken of the colorimetric response to each concentration and labelled (Figure 11b). A clear observable increase in color is apparent with the initial introduction of the lower concentration, however, as the concentrations increase, it proved harder to distinguish the concentrations present. This said, there was a clear orange color to all the samples containing amoxicillin.

**Figure 12:**
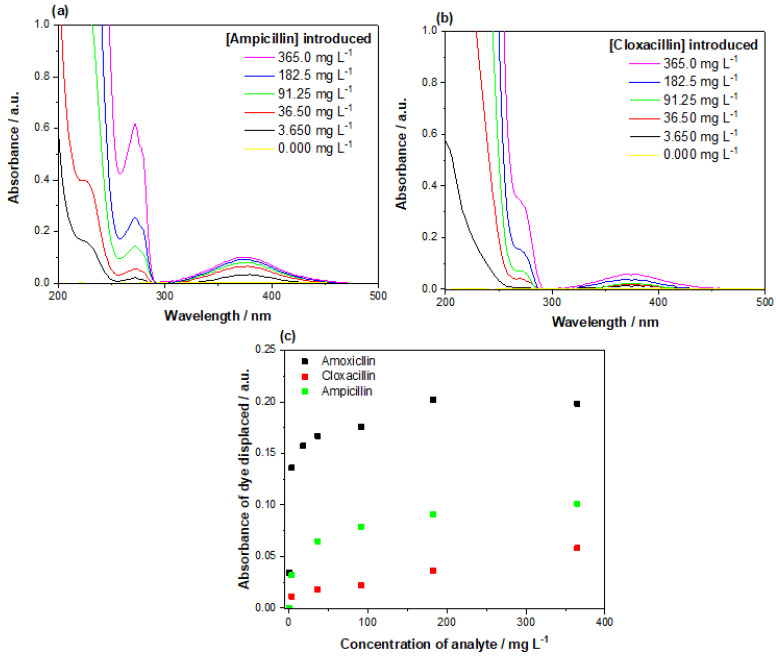
Dose response of the displacement assay towards (**a**) ampicillin and (**b**) cloxacillin, with the (**c**) maximum absorbance of the mordant orange dye (λ = 385 nm) plotted against the concentration of analyte present for comparison.

Alongside the response of the assay to amoxicillin, the selectivity of the assay was assessed with increasing concentrations of ampicillin and cloxacillin (Figure 12). Of the two aminopenicillins, ampicillin exhibited a stronger response when incubated with the assay, though the absorbance recorded overall was half that of the amoxicillin. Cloxacillin demonstrated a weaker interaction with the assay; though dye was still displaced, it was at a much lower concentration than what was observed for the other aminopenicillins tested. Overall, the reaction of the assay to the amoxicillin is much greater, though the selectivity of the assay clearly extends in a lesser extent towards compounds of the same class.

## 4. Discussion

### 4.1. Bulk Polymerization MIP Analysis

Of the compositions tested, the methacrylic acid/ethylene glycol dimethacrylate co-polymer proved to be the most efficient at binding the imprinted amoxicillin. The methacrylic acid has potential to interact with the amine, carboxylic acid, and hydroxyl functionalities within the molecule, offering multiple binding modes between the template and polymer. The acrylamide based MIP 204 did not share the same affinity as its MAA-based counterpart, with the trend of the MIP data being poor across the concentration range (hence the data was not fit). The reasoning for this could lie in the fact the carboxylic acid group is too easy to access on the edge of the molecule and therefore the imprints formed do not capture the full chemical architecture. Thus, the difference between MIP and NIP is diminished as the non-specific acrylamide at the surface of the NIP is just as likely to interact as the shallow imprints of the MIP making a trend harder to correlate. MIP 202 replaced the EGDMA with TRIM, yielding a more crosslinked polymeric structure stemming from the increased number of double bonds (3) that TRIM contains compared to that of EGDMA (2). The increased crosslinking provides extract mechanical rigidity to the polymer produced, though this had little impact on performing the overall performance of the MIP. The binding capacity was increased (12.10 μmol g^−1^), but the binding of amoxicillin at low concentrations (C_f_ = 0.1 mM) remained poor compared to that of the NIP. In contrast, MIP 203 had a slightly diminished binding capacity (9.47 μmol g^−1^), but performed well at low concentrations when compared to the NIP. This is reflected in the IF values calculated for the MIPs, where it is clear that MIP 203 has the superior specificity towards the amoxicillin over the other MIPs. When analyzing the data at higher C_f_, it is apparent that MIP 202 has increase in performance when compared to the NIP, whereas MIP 203 performances begins to slowly diminish. If the IF values are extrapolated and calculated at C_f_ = 0.6 mM, MIP 202 and 203 have values of 2.08 (NIP: 5.79 µmol g^−1^, MIP: 12.07 µmol g^−1^) and 2.01 (NIP: 4.70 µmol g^−1^, MIP: 9.44 µmol g^−1^) respectively. The convergence of the IF values indicates that the MIPs actually perform comparably at the higher concentrations and their performance is harder to differentiate. As antibiotics tend to be present at lower concentrations, it is logical to select the MIP that performs greater at lower concentrations, and therefore MIP 203 is the composition of choice.

These results are, however, surprising when comparing the data to the literature, as research found on the development of MIPs towards amoxicillin suggests that acrylamide provides the better binding solution [42]. However, the two acrylamide based MIPs tested in the research outline performed extremely poor when compared to that of the MAA-based MIPs. This difference in binding may be derived from the acidity/basicity of the solution used during the rebinding of the molecule. Amoxicillin is observed to be slightly acidic, offering potential to protonate the amine functionality and provide strong ionic interactions with the MAA in the developed polymers [43]. By altering the solution to be more basic nature, the acidic functionalities in the molecule could be stripped of their protons, facilitating ionic bond formation between the carboxylic functionalities and acrylamide in a MIP.

The general particle morphology for the bulk imprinted MIPs proved to be very heterogeneous, though this is to be expected as bulk imprinting involves the generation of polymeric monolith that is then ground into a fine powder [44]. The large discrepancy in particle shape and size yields poor performing MIPs, reducing MIP reproducibility in intra and inter sample analysis as particles sizes differ so vastly.

### 4.2. Emulsion MIP Structure

Emulsion polymerization was utilized in an attempt to increase the homogeneity in observed particle sizes and morphologies. As emulsion polymerization combines two phases to form an emulsion between an organic and aqueous medium, it is an ideal tool to produce more uniform particles [45]. In theory, there is little difference between monolithic bulk polymerization and emulsion polymerization, bar the use of water and a surfactant to generate small microspheres in comparison to a monolithic polymer [46]. The incorporation of a surfactant (SDS) helps stabilize the formed emulsion, producing micelles containing the lipophilic components (monomer, crosslinker, initiator) of the reaction and therefore acts as a small reactor that enables localized polymerization without it propagating throughout the whole solution [45]. The literature does, however, show that the surfactant and presence of water can have negative effects on the binding capabilities of the MIP [47], though there is opposing literature that suggests the presence of water may in fact help the formation and function of the MIP [48]. It is to be noted that different interactions occur during each process, and the purpose of the research is to see how generating more homogenous smaller particles by emulsion polymerization compare to a crushed monolith of the same composition. This said, the size of the micelles formed still depends on how well mixed the organic and aqueous phases are, therefore, an investigation into how the stirring affected the achieved particle sizes was conducted. Another consideration lies in the fact that the monolithic bulk composition was directly transferred into an emulsion type setting, which means that DMSO was still used in the organic phase as a porogen. DMSO is miscible with water that could prove problematic for the formation of micelles, though the other components present are less soluble and still enable micelle formation. There has been research conducted on systems that used DMSO as an alternative solvent for micelle formation when aqueous conditions would not be feasible [49]. It has been shown that micelles still form due to the high polarity of DMSO and a surfactant can still function to form micelles at the phase boundaries between polar and non-polar interfaces [50]. In the case of the generation of MIPs in this research, it could be speculated that DMSO would distribute between the water and less soluble components of the mixture, making it present inside and outside the micelle formation. However, a further study would have to be conducted to thoroughly investigate the true mechanism in this circumstance.

An overhead stirrer was chosen for the reaction process, as it provided greater control over the speed the mixture was subjected to, and ensured that the whole mixture received the same level of stirring compared to that of a magnetic stirrer. Ensuring different rpm could successfully be applied across the solution and a range of 600–1400 rpm was selected. Agitation at the lower speeds yielded larger particles (50–100 μm) with higher standard deviations, whereas higher speeds produced small particles (5–30 μm) with a smaller deviation. When plotting the particle size against rpm, a clear relation is viewed, with the particle sizes achieved plateauing above 1000 rpm. Though the particle sizes differs with rpm, the particle morphology does not. Optical microscope images reveal that the spherical morphologies of the particles are consistent across the speeds tested. Thus, the emulsion polymerization demonstrates a visible increase in the homogeneity of particle sizes and structure when compared to that of the bulk polymerization method.

### 4.3. Binding of Emulsion MIP

When analyzing the emulsion MIP 205 data, it is clear that it possess a higher binding capacity (22.53 μmol g^−1^) towards amoxicillin when compared to the monolithic bulk generated particles (9.89 μmol g^−1^). The increased homogeneity of the particles in MIP 205 are the clear source of this increased capacity, providing smaller, more uniform particle sizes that facilitate greater mass transfer and surface area when compared to the roughly ground particles [51]. MIP 205 performs greatly at lower concentrations (below C_f_ = 0.15 mM) and quickly saturates, whereas MIP 203 performs across the full concentration range. The IF values compared in Table 3 relates the performance of the MIPs at a defined free concentrations (C_f_ = 0.1), analyzing the specific interaction of the MIPs more clearly. These IF values compliment Figure 8, giving a clear metric of how much greater the interaction with MIP 205 is compared to MIP 203. It can be speculated that the loss in performance at higher concentrations is a direct result of simply transferring monolithic bulk optimized components to the emulsion methodology, or as previously mentioned the presence of water and surfactant during polymerization, reducing the binding affinities at the higher concentrations. However, this drop in performance at higher concentrations is inconsequential as the concentration range for antibiotics within wastewater, effluent, and/or milk are much lower (286.7–909 ng L^−1^) in concentration [52,53].

Exposure of the MIP 205 to other antibiotic analogues (ampicillin and cloxacillin) reveal that the MIP performs well at binding cloxacillin, whereas it poorly binds ampicillin. Between the two analogues tested, ampicillin holds the closer resemblance to amoxicillin, though the structure lacks the presence of the hydroxyl functionality of the terminal aromatic system. The absence of this group seems to critically affect the binding of ampicillin, with a diminished maximum binding capacity of 4.71 μmol g^−1^ observed. Loss of the strong hydrogen bond acceptor/donor functionality seems the rationale for this, being in an easily accessible sterically unhindered position [54]. Cloxacillin does not display the same reduced binding capacity (16.43 μmol g^−1^), despite its much larger molecular weight and bulk chemical composition. The structure does, however, retain the presence of the oxygen heteroatom alongside an additional nitrogen, which could be the argument for this. The addition of the nitrogen and subsequent lone pair of electrons would facilitate extra binding via a hydrogen donor interaction, and even an ionic interaction as the functionality becomes positively charged by accepting a proton [54].

The specific interaction (IF) of each analogue was once again evaluated at C_f_ = 0.1 mM to enable a direct comparison of each of the compounds (Table 3). When analyzing these values, it is clear that amoxicillin has the highest IF value by quite some way, though now it is observed that ampicillin actually has the higher specific interaction when compared against cloxacillin. This may be down the aforementioned reasons with ampicillin being closer to the structure of amoxicillin, so therefore can bind more specifically, whereas cloxacillin has more groups that can provide non-specific interactions. Though the intended sensor is developed towards amoxicillin, there is an argument to be made that a broader sensor for aminopenicillins as a whole is more preferable as it would facilitate broad spectrum testing of samples. Therefore, the indicated affinity of the sensor towards cloxacillin could be of benefit depending on the setting the sensor is deployed in.

### 4.4. Selection of a Dye for Displacement

Malachite green and crystal violet were selected for testing as potential dyes to be displaced based on their previous use in other research [26,27]. Their chemical structures contained terminal amine functionalities that offer ionic bonding to the methacrylic acidic functionalities in the MIP. The observed binding capacity of malachite green and crystal violet reflect this, with binding capacity surpassing that of amoxicillin (28.33 μmol g^−1^ and 49.73 μmol g^−1^). The increased interactions between the dyes and MIP can stem from the increased number of easily accessible amine functionalities present, and/or the relative size of the molecules when compared to that of amoxicillin [55]. Mordant orange on the other hand contains an azo functionality placed between two aromatic systems, making it more hindered and harder to interact with. This fact is reflected in the maximum binding capacity observed (24.18 μmol g^−1^), though mordant orange possess a nitro and carboxylic acid functional that can provide additional interactions with the MIP.

Previous research has demonstrated that there is a relationship between the imprint factor of the dye being displaced and the imprint factor of the target molecule displacing said dye [26,27]. For dye displacement to be successful, the IF value of the dye must be lower than that of the template molecule at the defined free concentration. Both malachite green and mordant orange have a lower IF value than amoxicillin at the lower free concentrations, making them both potentially suitable as dyes in the displacement assay. However, the prior research also suggests that the dye molecule must have a somewhat similar binding capacity to the MIP as the template molecule; otherwise, the dye would be less easily displaced. Out of these two dyes, mordant orange has the binding capacity more similar to that of amoxicillin, whereas malachite green is observed to have a higher maximum binding capacity that would not bode well for the displacement mechanism. Therefore, of the two molecules, mordant orange is the clear choice for the displacement assay.

### 4.5. Dye Displacement Assay

The dose response revealed that an observable displacement of dye was apart when the assay was incubated with 0.365 mg L^−1^, being clear on the UV-spectra and by analysis with the naked eye. After this point, the inspection of the color of the filtrate proved more difficult as the color gradient between samples is not always obvious. This said, a clear distinction between absorbance is seen in the UV-spectra, with the amount of dye displaced correlating to the amount of amoxicillin present. Normally, other organic pollutants are expected to present, which could mask/interfere (absorb at a same or similar wavelength) with the amoxicillin absorbance peak; therefore, it is beneficial having the higher wavelength dye present that is less likely to experience interferences/masking [56]. Past 91.25 mg L^−1^, however, there is little change in the amount of dye displaced, meaning higher concentrations of amoxicillin could not be correlated with a specific absorbance of dye. These results lend into the assay being used in a semi-quantitative manner, enabling the user to visually differentiate between low and high concentrations. The linear range unfortunately is lacking, which would impede the sensor in an environment where quantitation of the presence of amoxicillin is necessary.

Selectivity tests on the assay revealed that the assay is also sensitive towards the presence of ampicillin and cloxacillin. Figure 12a shows that ampicillin elicits a higher amount of dye displacement when compared to cloxacillin, though this amount is still only half the amount that the amoxicillin achieves. In the context of waste analysis for antibiotics, it would be fruitful to have an assay that is capable of detecting a wider range of antibiotics, compared to just a singular structure. The selectivity of the assay towards other aminopenicillins can therefore be seen as a positive, opening the sensor to the possibility of broad range analysis across the class of compounds. As previously discussed, it is logical that other compounds of the same class interact with the assay as they did with the MIP. If the assay was to be developed further towards the sole purpose of detecting amoxicillin, then the selectivity of the MIP must be improved to remove the cross selectivity observed in the assay. This may, however, prove troublesome as aminopenicillins share very similar chemical architectures, making it more sensible to expand the capabilities of the MIP towards class determination rather than a compound specific analysis.

## 5. Conclusions

Overall, the research demonstrates how a colorimetric assay for the detection of amoxicillin can be easily generated in just a few steps. The work utilizes simple bulk MIPs to discover polymeric compositions that interact with amoxicillin and builds these findings into a more refined MIP morphology that improves sensitivity and homogeneity by means of emulsion polymerization. During the synthesis of these more refined particles, a relationship between the speed of agitation and particle sizes produced was observed. This relationship could be exploited to make more reliable MIP sizes on a larger scale that holds both academic and industrial value. These more homogeneous particles demonstrated high specificity towards amoxicillin at low concentrations (C_f_ = 0.1 mM), while proving selective and showing minimal interaction with compounds of the same class (ampicillin and cloxacillin). Alongside aminopenicillin analogues, various dyes (malachite green, crystal violet, and mordant orange) were tested for their specific binding towards the MIP and their interactions studied. Malachite green and crystal violet have been previously used in literature to produce dye displacement assays, though mordant orange has never been tested for such a possibility. This research, therefore, pushes the boundaries that are currently in place for the selection of dyes for these kind of MIP-based dye displacement assays. Once the optimum dye was identified, the developed MIP was easily converted into a visual sensor by the simple loading of mordant orange that could easily be displaced in the presence of the template molecule—amoxicillin.

Selectivity tests conducted over a wider concentration regime display that other aminopenicillins could give a false positive reading, particularly at higher concentrations. Although many antibiotic-detection kits suffer from the same drawbacks and some applications might actually ask for antibiotic class detection rather than specifically identify the template compound, the current results illustrate that the selectivity needs to be further fine-tuned for application in complex samples. At this point in its development, the sensor is mainly suitable as an easy and fast method to quantify amoxicillin in pure samples.

Overall, the novelty of the research lies in the fact that this is the first time an MIP-based dye displacement assay has been generated for amoxicillin using this format with the combination of mordant orange. The findings from this research has potential to be used in both a quantitative and semi-quantitative manner depending on if analytical apparatus is on hand. The key technical advantage being shorter analysis time, no sample pre-treatment, and portability. In short, the methods outlined in this paper could be utilized in the rapid analysis of aqueous samples for the unwanted presence of the antibiotic amoxicillin.

## Figures and Tables

**Table 1:** MIP/NIP compositions.

MIP/NIP	Methodology	Monomer	Crosslinker	Initiator	Solvent	Template
MIP 201	Bulk	AA	TRIM	AIBN	DMSO	Amoxicillin
NIP 201	Bulk	AA	TRIM	AIBN	DMSO	-
MIP 202	Bulk	MAA	TRIM	AIBN	DMSO	Amoxicillin
NIP 202	Bulk	MAA	TRIM	AIBN	DMSO	-
MIP 203	Bulk	MAA	EGDMA	AIBN	DMSO	Amoxicillin
NIP 203	Bulk	MAA	EGDMA	AIBN	DMSO	-
MIP 204	Bulk	AA	EGDMA	AIBN	DMSO	Amoxicillin
NIP 204	Bulk	AA	EGDMA	AIBN	DMSO	-
MIP 205	Emulsion	MAA	EGDMA	AIBN	DMSO/H_2_O	Amoxicillin
NIP 205	Emulsion	MAA	EGDMA	AIBN	DMSO/H_2_O	-

**Table 2:** Binding capacities (S_b_) for MIP 201, 202, 203, and 204 at C_f_ = 0.1, 0.2, and 0.3 mM, and their corresponding imprint factors (IF). Values were drawn directly from the fitted curve of each MIP/NIP at the defined concentration.

MIP/NIP	S_b_/μmol g^−1^ (at C_f_ = 0.1 mM)	IF (at C_f_ = 0.1 mM)	S_b_/μmol g^−1^ (at C_f_ = 0.2 mM)	IF (at C_f_ = 0.2 mM)	S_b_/μmol g^−1^ (at C_f_ = 0.3 mM)	IF (at C_f_ = 0.3 mM)
201	MIP 5.07 NIP 2.82	1.80	MIP 7.29 NIP 5.38	1.35	MIP 8.80 NIP 6.41	1.37
202	MIP 4.33 NIP 2.65	1.63	MIP 9.74 NIP 5.00	1.95	MIP 11.36 NIP 5.62	2.02
203	MIP 6.36 NIP 2.49	2.55	MIP 8.11 NIP 3.27	2.48	MIP 8.93 NIP 3.83	2.33
204	N/A	N/A	N/A	N/A	N/A	N/A

**Table 3:** Binding capacities (S_b_) for MIP 203 (amoxicillin, ampicillin, and cloxacillin) and MIP 205 at C_f_ = 0.1 and their corresponding imprint factors (IF). Values were drawn directly from the fitted curve of each MIP/NIP at the defined concentration.

MIP	Compound	S_b_/μmol g^−1^ (at C_f_ = 0.1 mM)	IF (at C_f_ = 0.1 mM)
203	Amoxicillin	MIP 6.36 NIP 2.49	2.55
205	Amoxicillin	MIP 21.45 NIP 0.47	45.64
205	Ampicillin	MIP 0.39 NIP 0.28	1.39
205	Cloxacillin	MIP 8.58 NIP 7.80	1.1

**Table 4:** Binding capacities (S_b_) for malachite green, crystal violet, and mordant orange at C_f_ = 0.1 mM, and their corresponding imprint factors (IF). Values were drawn directly from the fitted curve of each MIP/NIP at the defined concentration.

Compound	S_b_/μmol g^−1^ (at C_f_ = 0.1 mM)	IF (at C_f_ = 0.1 mM)
Malachite green	MIP 19.09 NIP 15.22	1.25
Crystal violet	MIP 36.92 NIP -	-
Mordant orange	MIP 7.31 NIP 3.77	1.94

## Data Availability

The data presented in this study are available in the document and Appendix A (Figure 1, Figure 2, Figure 3, Figure 4, Figure 5, Figure 6, Figure 7, Figure 8, Figure 9, Figure 10, Figure 11 and Figure 12).
